# Effects of Platelet-Rich Plasma in Tear Size Reduction in Partial-Thickness Tear of the Supraspinatus Tendon Compared to Corticosteroids Injection

**DOI:** 10.1186/s40798-023-00556-w

**Published:** 2023-02-08

**Authors:** Thanathep Tanpowpong, Marvin Thepsoparn, Numphung Numkarunarunrote, Thun Itthipanichpong, Danaithep Limskul, Phark Thanphraisan

**Affiliations:** 1 Department of Orthopaedics, Faculty of Medicine, Chulalongkorn University, King Chulalongkorn Memorial Hospital, The Thai Red Cross Society, Bangkok, Thailand; 2 Pain Management Research Unit, Department of Anesthesia, Faculty of Medicine, Chulalongkorn University, King Chulalongkorn Memorial Hospital, The Thai Red Cross Society, Rama IV Rd, Bangkok, 10330 Thailand; 3 Department of Radiology, Faculty of Medicine, Chulalongkorn University, King Chulalongkorn Memorial Hospital, The Thai Red Cross Society, Bangkok, Thailand

**Keywords:** Platelet-rich plasma, Magnetic resonance imaging, Tendinopathy, Adrenal cortex hormones steroids, Pain

## Abstract

**Objectives:**

Corticosteroid (CS) injection is commonly used in partial-thickness rotator cuff tears to decrease pain. However, this could result in unwanted side effects, such as tendon rupture. Alternatively, platelet-rich plasma (PRP) injection is frequently used to treat tendinopathies because it enhances healing. This study aimed to compare the differences in tear size and functional scores between intralesional PRP and subacromial CS injections.

**Methods:**

Patients with symptomatic partial-thickness tears of the supraspinatus tendon who underwent conservative treatment for ≥ 3 months were enrolled. All patients underwent magnetic resonance imaging (MRI) to confirm the diagnosis. Fourteen and 15 patients were randomized to receive intralesional PRP and subacromial CS injections, respectively. Tears were measured in the coronal and sagittal planes. The patients underwent another MRI 6 months after the injection. Tear size was compared between the two MRI results. The American Shoulder and Elbow Surgeons Shoulder score (ASES) and Constant–Murley score (CMS) were also obtained.

**Results:**

The baseline data were similar between the groups. In the coronal plane, PRP and CS showed tear size reductions of 3.39 mm (*P* = 0.003) and 1.10 mm (*P* = 0.18), respectively. In the sagittal plane, PRP and CS showed tear size reductions of 2.97 mm (*P* = 0.001) and 0.76 mm (*P* = 0.29), respectively. Functional scores improved 6 months after injection in both groups, but PRP showed better functional scores than CS (*P* = 0.002 for ASES, *P* = 0.02 for CS).

**Conclusion:**

Intralesional PRP injection can reduce the tear size in partial-thickness tears of the supraspinatus tendon. Subacromial steroid injection did not significantly affect the tear size. While CS improved functional scores compared with baseline, PRP resulted in better improvement 6 months post-injection.

*Trial registration* Thai Clinical Trials Registry, TCTR20210428004. Registered 28 April 2021-retrospectively registered, TCTR20210428004.

## Key points

- Intralesional platelet-rich plasma injection reduced the tear size in partial tears of the supraspinatus tendon, while subacromial steroid injection did not affect the tear size.
- While steroid improved functional scores compared with baseline, platelet-rich plasma injection resulted in better improvement at 6 months after injection.
- Platelet-rich plasma seems to be a good alternative for treating supraspinatus tendon tears.

## Introduction

A partial tear of the supraspinatus tendon results in pain and dysfunction [1, 2]. In symptomatic patients, the pain can be relieved nonoperatively by corticosteroid (CS) injection. CS injection is commonly used in partial-thickness rotator cuff tears for symptomatic pain relief because of its cost-effectiveness and feasibility [3]. However, the effectiveness of the pain relief is short, and long-term function is not improved [3–5]. The injection could also lead to unwanted side effects, such as tendon rupture [6, 7]. Furthermore, CS injection was found to be associated with an increased risk of revision surgery in those who underwent rotator cuff repair [8, 9].

Recently, the use of biologic augmentation, such as platelet-rich plasma (PRP), has gained popularity for the treatment of tendon pathologies, and the ability of PRP to enhance healing has led to its increased use [10–12]. Magnetic resonance imaging (MRI) is the imaging modality of choice for determining the tear size of the rotator cuff [13]. The modality has high accuracy and is not invasive; therefore, performing an MRI study as a follow-up is deemed feasible. In a recent study, the tear of the supraspinatus tendon was followed up with ultrasound imaging [11, 14]; however, the results of ultrasound are inconsistent and operator dependent. In some other studies comparing CS and PRP injections, tendon tears were grossly classified and compared using imaging modalities [12, 15], in which healing could not be well observed. Owing to the variation of the location of partial-thickness tears, subacromial PRP injection may not effectively enhance healing. We hypothesized that intralesional PRP injection using ultrasound guidance in partial-thickness supraspinatus tears would directly stimulate healing at the tear site, thereby decreasing the tear size measured by MRI.

The primary objective of this study was to measure the difference in tear size before and after CS compared with PRP injections. The secondary objective of this study was to measure functional outcomes before and after injection.

## Methods

**Fig. 1:**
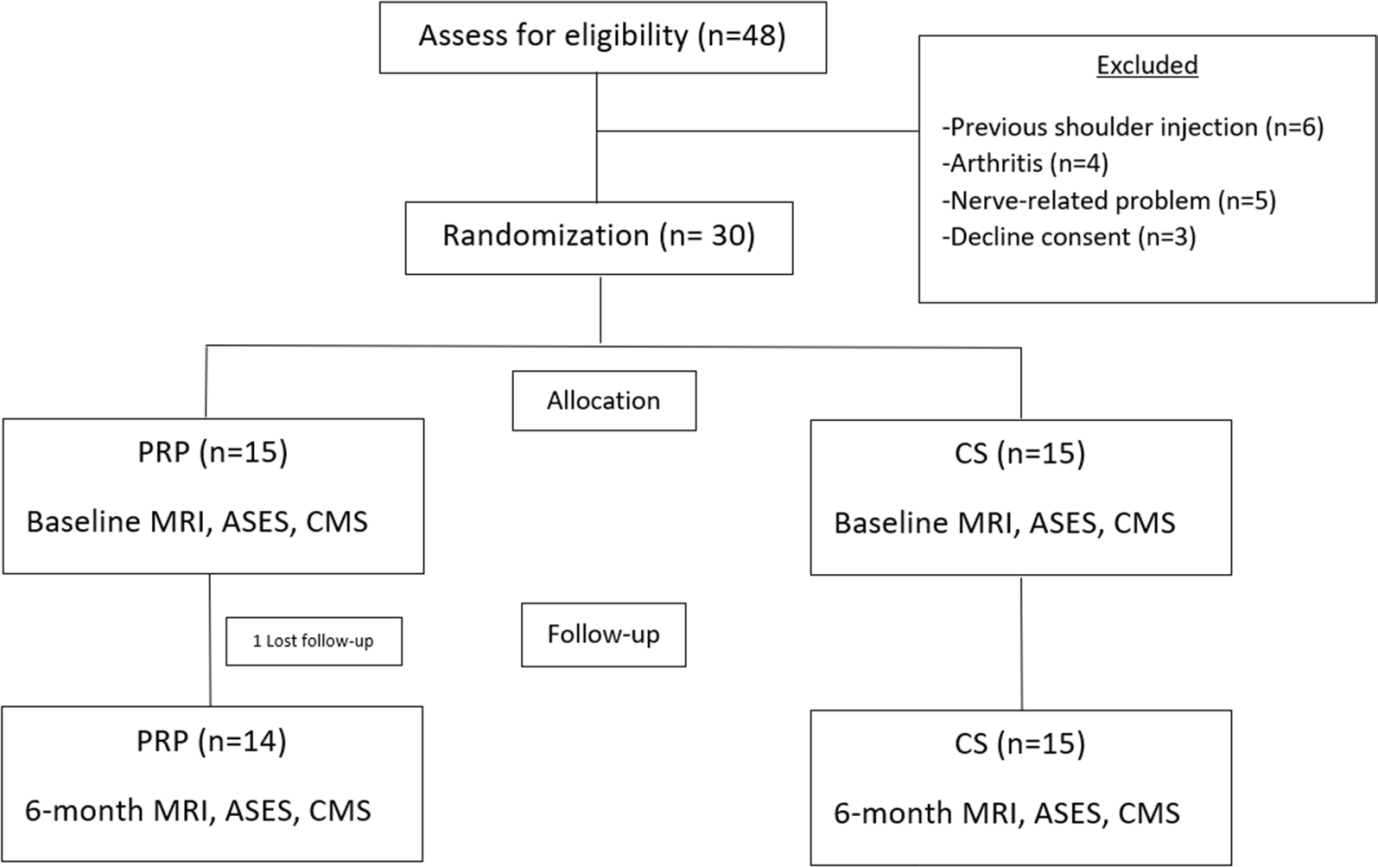
Consolidated Standards of Reporting Trials (CONSORT) diagram. PRP, platelet-rich plasma; CS, corticosteroid; ASES, American Shoulder and Elbow Surgeons Shoulder score; CMS, Constant–Murley score

This study was conducted between May 2020 and March 2021 at the Faculty of Medicine, Chulalongkorn University, King Chulalongkorn Memorial Hospital and Thai Red Cross, Bangkok, Thailand. This single-center randomized controlled trial was approved by the institutional review board (IRB No.230/64, TCTR20210428004) and was done under strict CONSORT guideline (Fig. 1). We enrolled patients with painful partial-thickness tears of the supraspinatus tendon, which was clinically diagnosed by an orthopedist (T.T.) with more than 10 years of experience. Thereafter, an MRI study of the affected shoulder was interpreted by a musculoskeletal radiologist (N.N.) with more than 10 years of experience with a corresponding radiologic diagnosis.

The shoulder MRI was performed using dedicated shoulder coils and either 1.5-T or 3-T magnets (Ingenia, Philips Medical System, Best, the Netherlands). Patients were in supine position with the arm rotated externally during imaging. The following MRI protocols were used: axial and coronal oblique proton density (TR/TE, 2500/25 ms); axial, coronal oblique and sagittal oblique fat-suppressed T2-weighted (TR/TE, 3500–4000/60–65 ms); sagittal oblique T1-weighted (TR/TE, 500–600/15). The following imaging parameters were used: echo-train length, 14–18 on T2-weighted image and 3–6 on T1-weighted image and 10–14 on proton density; FOV 15 × 17 cm to 16 × 18 cm; matrix, 300 × 245–350 × 284; section thickness, 3.0–3.5 mm; and interslice gap , 0–0.5 mm.

### Inclusion and Exclusion Criteria

Eligible patients were at least 18 years old. The patients enrolled had undergone conservative treatments, including lifestyle modification, oral analgesics, or physical therapy for at least 3 months, with the exception of shoulder injection.

We excluded patients with arthritis or other related complications, such as infection of the glenohumeral joint; nerve-related problems, such as cervical radiculopathy; malignancy, coagulopathy, immunocompromised conditions; previous shoulder injection or surgery; history of trauma to the affected shoulder; or unwillingness to provide informed consent.

### Randomization

After informed consent was obtained from the patients, the patients were randomly assigned to the CS or PRP group using computer-generated block randomization. This number was assigned and concealed in an envelope. Group assignments were only accessible to research assistants and a pain specialist (M.T.) in the study and were concealed from the patients, orthopedist, and radiologist assessors. The patients and the assessors were blinded.

### Injection

All procedures were performed in an operating room. The injected substance was prepared once the pain specialist opened the envelope, which specified the group allocation. All injections were administered by a single experienced pain physician (M.T.). Patients sat in the modified Crass position [16], and the area to be injected was disinfected under strict aseptic protocols. The posterolateral approach was employed for all patients in both groups. Real-time ultrasound guidance (Phillips, Yokogawa Medical Systems, Tokyo, Japan) using a 3- to 12-MHz linear array transducer covered with a sterile camera sleeve in the plane technique was employed in all injections. The supraspinatus tendon and subacromial–subdeltoid bursa were identified. After the injection, there was a 30-min observation period for potential immediate complications, such as fever, chills, or pruritus.

For the CS group, the patients received triamcinolone acetonide suspension (Kenacort-A suspension, 40 mg/mL) mixed with 4 mL of 1% lidocaine. The solution was prepared using a 5-mL syringe with a 25-gauge needle and was injected into the subacromial bursa of the affected shoulder. This represents the injection commonly performed in practice.

For the PRP group, we used a double-syringe system (Arthrex ACP, Naples, FL, USA) for PRP extraction. Patients in the group had 15 mL of their blood drawn, which was then centrifuged at 1,500 revolutions per minute for 5 min. Once the centrifugation was complete, double-syringe extraction was performed, which resulted in 5 mL of leukocyte-poor (LP) PRP [17]. The freshly prepared LP-PRP was injected into the supraspinatus tendon tear sites using a 25-gauge needle within 5 s after centrifugation.

### Data Collection

**Fig. 2:**
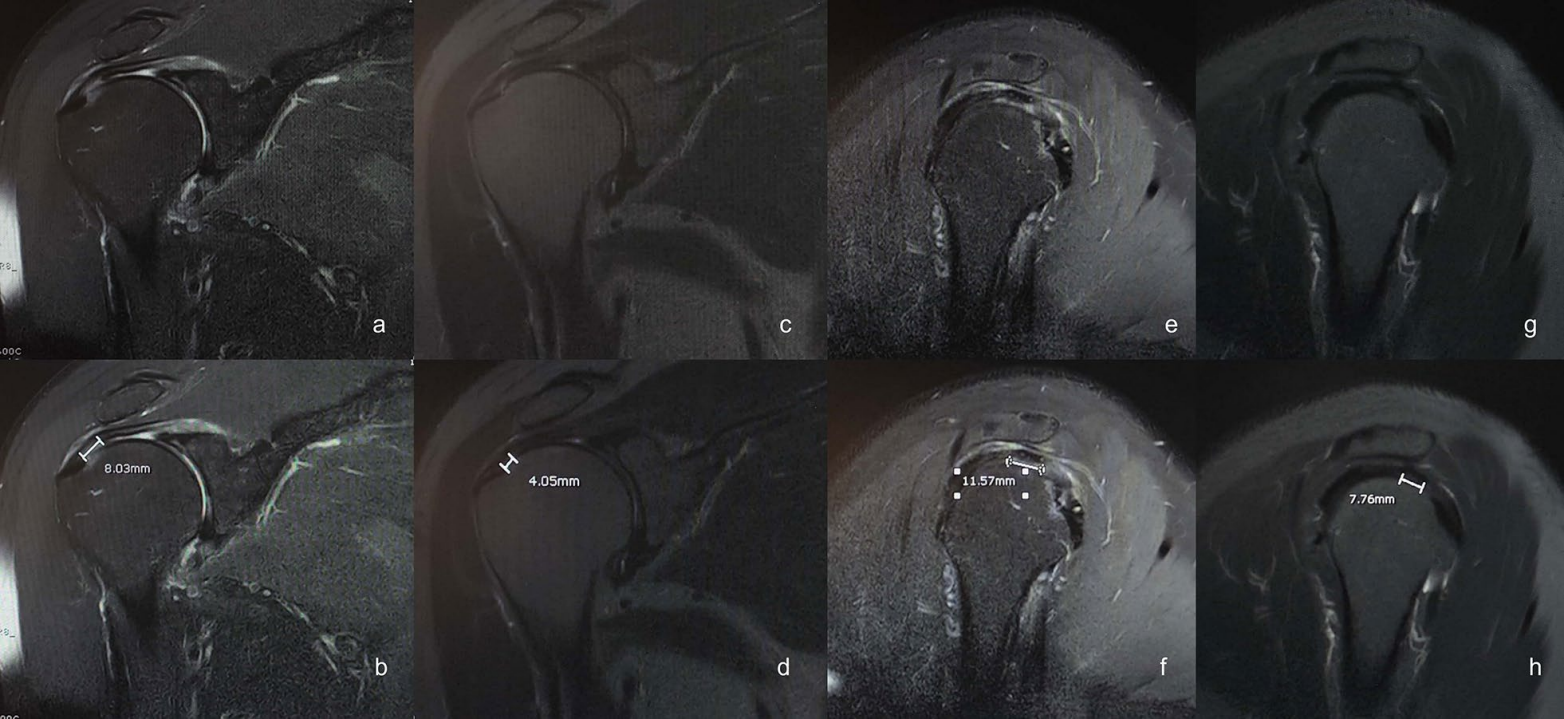
A coronal oblique MRI image of the right supraspinatus tendon tear (**a**) with measurement (**b**) before the injection. A coronal oblique MRI image of the right supraspinatus tendon tear 6 months after the injection (**c**) with measurement (**d**). A sagittal oblique MRI image of the right supraspinatus tendon tear (**e**) with measurement (**f**) before the injection. A sagittal oblique MRI image of the right supraspinatus tendon tear 6 months after the injection (**g**) with measurement (**h**)

Two functional scores, namely the American Shoulder and Elbow Surgeons Shoulder score (ASES) [18] and the Constant–Murley score (CMS) [19], were obtained before the injection at the time of diagnosis. Six months after the injection, MRI was performed again to evaluate the supraspinatus tendon tear. The radiologist and another orthopedic assessor (D.L.) measured the tear size and interpreted the results to observe the interrater correlation of the measurements (Fig. 2). For each assessor, the MRI images from before and after the injection were reviewed and measured twice, 1 month apart, to observe the intrarater correlation of the measurement. Functional scores were also calculated. The mean of the two measurements from both assessors was determined and used for further calculations. ASES and CMS were obtained from the patients at 6 months after the injection by the experienced orthopedist (T.T.).

### Statistical Analysis

For sample size determination, we used a statistical power of 80% and a two-sided significance level of 0.05. Based on previous data [20], with the intention to detect the difference in tear size at 6 months, we enrolled 15 patients for each group, accounting for a 10% loss to follow-up. Shapiro–Wilk test was used for testing normality of data distribution. Levene’s test for equality of variance was done. Pearson’s correlation was done to observe tear size reduction and functional score improvement.

SPSS (version 22.0; SPSS Inc., Chicago, IL, USA) was used for the statistical calculations. An unpaired t-test was used to compare tear size, ASES, and CMS between the two groups. A paired t-test was used to compare the differences between the groups. Patient data were compared using the unpaired t-test, Chi-square test, and Fisher exact test. Tear type was analyzed using the Fisher exact test. Statistical significance was set at *P* < 0.05.

For tear size measurement, the intraclass correlation coefficient was used for the interrater and intrarater reliability tests. The scoring system from Fleiss et al. [21] was used to interpret the results (good > 0.75, fair 0.4–0.75, poor < 0.4).

### Patient Involvement

All patients were provided information regarding the details of the study and time required to participate in the study. The patients did not participate in designing the study or result interpretation process.

## Results

We enrolled 30 patients in this study, and these patients were randomized into two groups. There were no significant differences between the two groups in terms of sex, age, affected side, affected dominant shoulder, tear type, tear size, or functional scores at baseline (Table 1). One patient in the PRP group was excluded from the study because we could not contact the patient for the second MRI, and the reason for the loss to follow-up was unknown.

**Table 1:** Baseline data of the two groups

Parameter	PRP [14]	CS [15]	*P*-value
Age (years)	58.31 (10.70)	63.27 (11.03)	0.24
Sex (male/female) (n)	02:12	03:12	> 0.99
BMI (kg/m^2^)	21.19	22.12	0.21
Affected side (left/right) (n)	09:05	05:10	0.14
Affected dominant side	57.14%	66.67%	0.71
Tear (n)			
Articular	8	5	0.36
Bursal	6	9	
Interstitial	0	1	
Tear size (mm)			
Coronal	7.96 (1.59)	7.02 (2.32)	0.27
Sagittal	6.80 (2.76)	5.87 (1.59)	0.22
Functional scores			
ASES	29.29 (8.45)	27.32 (8.58)	0.74
CMS	31.79 (8.38)	23.43 (9.54)	0.24

Age, tear size, and functional scores are represented as mean (standard deviation)

*PRP* platelet-rich plasma, *CS* corticosteroid, *ASES* American Shoulder and Elbow Surgeons Shoulder score, *CMS* Constant–Murley score

### Tear Size

**Fig. 3:**
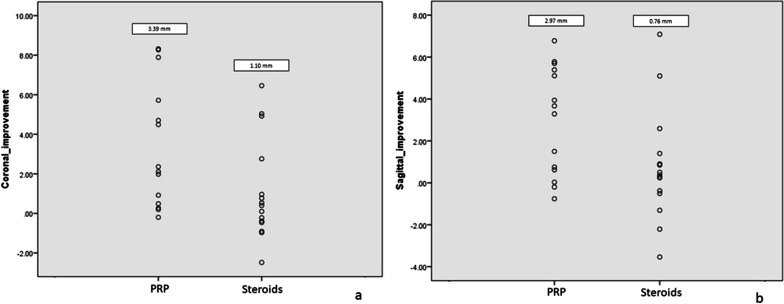
Dot plots show tear size reduction in the coronal plane (**a**) and sagittal plane (**b**) after PRP and steroids injection

The tear sizes before and after injections were tested to be normally distributed. At the 6-month follow-up, the tear size of the PRP group in the coronal plane was significantly decreased (3.39 mm; *P* = 0.003), compared with the nonsignificant tear size reduction in the CS group (1.10 mm; *P* = 0.18). There was a significant difference in tear size reduction between the two groups (2.29 mm; 95% confidence interval [CI], 0.32 to 4.47) (Fig. 3).

In the sagittal view, the tear size of the PRP group was significantly decreased (2.97 mm) compared with the baseline (*P* = 0.001), while a nonsignificant tear size reduction was observed in the CS group (0.76 mm; *P* = 0.29). There was a significant difference in tear size reduction between the two groups (2.21 mm; 95%CI, 0.37 to 4.21) (Table 2) (Fig. 3).

**Table 2:** Tear size of the two groups measured by MRI

Tear size (mm)	PRP	CS	Difference (95% CI)
Coronal			
Baseline	7.96 (1.59)	7.02 (2.32)	
6 months	4.57 (2.05)	5.92 (1.96)	
Tear size reduction	3.39	1.1	2.29 (0.32 to 4.47)
*P*-value	0.003	0.18	0.04
Sagittal			
Baseline	6.80 (2.76)	5.87 (1.59)	
6 months	3.83 (1.80)	5.11 (1.71)	
Tear size reduction	2.97	0.76	2.21 (0.37 to 4.21)
*P*-value	0.001	0.29	0.03

The baseline and 6-month tear size values are presented as mean (standard deviation)

*PRP* platelet-rich plasma, *CS* corticosteroid, *CI* confidence interval

The MRI measurements were evaluated for intrarater correlation for each assessor and for interrater correlation between the assessors. The measurements had good intrarater and interrater correlations at the baseline and 6-month MRI tests (Table 3).

**Table 3:** Intraclass correlation coefficients (interrater and intrarater) of the MRI measurements by the two assessors

	Intrarater assessor 1	Intrarater assessor 2	Interrater
- Baseline	0.87	0.84	0.79
- 6 months	0.93	0.83	0.76

### Functional Scores

**Fig. 4:**
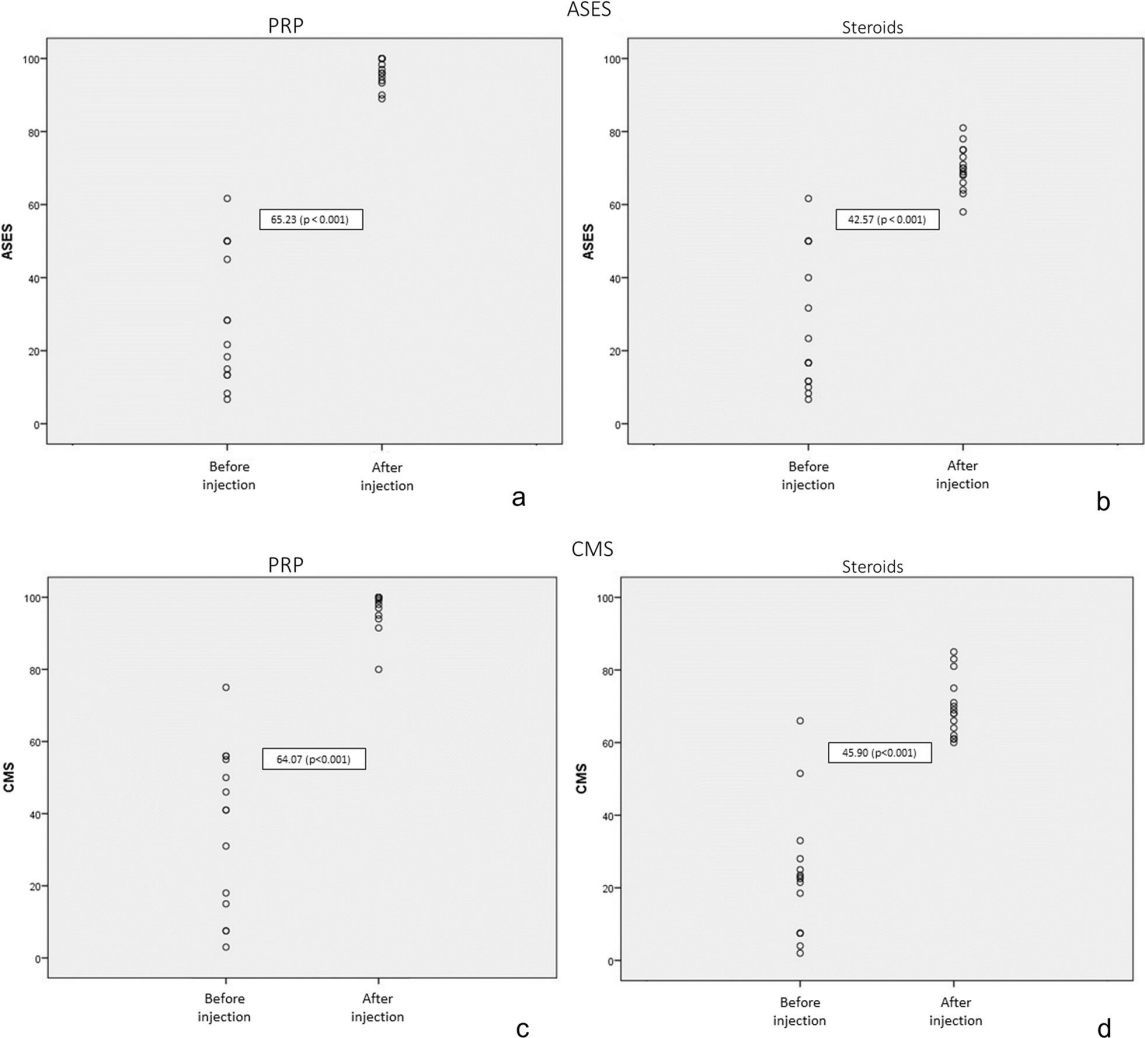
ASES comparison before and 6 months after injection of PRP (**a**) and steroids (**b**). CMS comparison before and 6 months after injection of PRP (**a**) and steroids (**b**). ASES, American Shoulder and Elbow Surgeons Shoulder score; CMS, Constant–Murley score

In terms of functional scores of the shoulder, significant increases in both ASES and CMS were observed in the PRP and CS groups at 6 months. At 6 months, the ASES was 94.52 (standard deviation, SD 7.05) for the PRP group and 69.89 (SD 5.53) for the CS group. There was a significant difference in ASES between the two groups at 6 months (24.63; *P* = 0.002). The CMS at 6 months was 95.86 (SD 6.03) for the PRP group and 69.33 (SD 8.64) for the CS group. There was a significant difference in CMS between the two groups at 6 months (26.53; *P* = 0.02) (Fig. 4).

Pearson’s correlation was done to see whether there was a correlation between tear size reduction and functional score improvement. There was no correlation between tear size reduction in the coronal plane and the ASES (*r* = 0.04) and CMS (*r* = 0.04). The reduction in tear size in the sagittal plane also did not correlate with the ASES (*r* = 0.19) and CMS (*r* = 0.14). However, we found that there was a correlation between tear size reduction between the coronal and sagittal plane (*r* = 0.71, *P* < 0.001).

## Discussion

### Tear Size

This study revealed a significant reduction in tear size in the PRP group compared with the CS group in the coronal and sagittal views. Only a few studies have focused on tear size after injection. Shams et al. [12] studied the effectiveness of the subacromial injection of PRP and steroid injections in symptomatic partial rotator cuff tears. The MRI results showed an overall nonsignificant improvement in the grades of tendinopathy in both groups. However, the MRI findings were graded on a 0 to 5 scale, and small differences may not be detected when the tear of interest is relatively small.

Ibrahim et al. [14] performed the ultrasound-guided injection of PRP and steroids for the treatment of rotator cuff tendinopathy. The study found an improvement in tendinitis, partial tear, and effusion at 2 months in the PRP group when compared with steroids. However, the tear size was not measured in their study. Niazi et al. [22] conducted a similar study and found an improvement in tear size at 6 months. Nevertheless, the tear size was evaluated by ultrasound imaging, and the measured value could have been affected by the ultrasound operator.

Cai et al. [23] evaluated healing after PRP injection for partial-thickness rotator cuff tears. PRP injection showed a decreased tear size of 2.89 mm anteroposteriorly at 12 months. We discovered similar results when evaluating tears in the sagittal plane. In a meta-analysis by Chen et al. [24] regarding the use of PRP for the improvement in rotator cuff tears, there was a significant reduction in long-term tears in PRP-treated patients. We believe that the significant reduction in tear size observed in the current study resulted from the PRP injection technique. With this technique, the concentrated PRP could directly cover the location of the torn tendon, whether the torn tendon was articular or bursal. To the best of our knowledge, this is the first study to directly compare MRI-measured tear sizes between the two injection methods.

The injected PRP, which resulted in a decreased tear size, could have resulted from inflammation which helped in tendon repair. Hudgens et al. [25] conducted a controlled laboratory study and reported that tendon fibroblasts with PRP activated the cellular TNF-α and NF-κB signaling pathways. This resulted in a transient inflammatory process, which could be responsible for the initiation of the tissue regeneration response. Regarding the type of PRP, Fitzpatrick et al. [26] conducted a meta-analysis and supported the use of a single injection of leukocyte-rich (LR) PRP under ultrasound guidance in tendinopathy. However, the number of LR-PRP studies is notably greater than that of LP-PRP. A study by Muthu et al. [27] comparing the efficacy and safety of LR-PRP and LP-PRP in the management of lateral epicondylitis concluded that both types offer similar results. In our study, we chose LP-PRP because of its availability and feasibility in conducting the research.

The injection of steroids did not increase tendon tear size, and we found no significant decrease in tear size after steroid injection. Liu et al. [20] performed intrasubstance steroid injection for full-thickness supraspinatus tendon tears and found no increase in tear size after the injection. Another study by Baverel et al. [9] found that preoperative CS injection had no influence on the retear rate after rotator cuff repair. These findings suggest that the injection is relatively safe and does not cause further harm to the tendon clinically because steroid injection is commonly used in practice.

### Functional Scores

We found notable improvements in both functional scores (ASES and CMS). Jo et al. [28] compared PRP and steroid injections for the treatment of rotator cuff disease and found better improvement in overall function and DASH (disabilities of the arm, shoulder, and hand) score but not in CMS after 6 months of PRP injection compared with steroids. Rossi et al. [29] conducted a similar study and found increased ASES and CMS after PRP injection. The study also found a good satisfaction outcome and rate of return to sports after the injection. Kwong et al. [11] conducted a similar study and found that ASES was higher in the PRP group when compared with the CS group at 3 months after the injection.

In contrast, Von Wehren et al. [15] found no significant difference between PRP and steroids after lateral subacromial injection at 6 months. A recent meta-analysis by Lin et al. [10] reported no significant changes in functional outcomes after PRP injection. It is difficult to conclude whether this result was due to the heterogeneity of the studies, especially when PRP is injected using different techniques.

### Limitations

First, it did not include a placebo control. However, many studies [3, 5, 6, 30] have found that steroid injection leads to better improvement in shoulder function, and pain relief was a concern for the patient in this study. Therefore, CS was chosen as a control in this study. Second, we did not consider cost-effectiveness. The much lower cost of the steroids and the feasibility of the injection might make steroids a more considerable alternative option in clinical practice. Third, this study had a relatively small sample size. A larger sample size should allow the demonstration of greater differences between the groups. However, a sample size calculation was done, and the calculated size was considered to be hypothetically sufficient.

## Conclusion

The intralesional injection of PRP in partial supraspinatus tendon tears reduced the tear size, while subacromial steroid injection did not significantly affect the tear size. Both PRP and steroid injection improved functional outcomes compared with baseline. Nevertheless, PRP injection resulted in better improvement 6 months after injection.

## Data Availability

The datasets generated during and/or analyzed during the current study are available from the corresponding author on reasonable request.
